# Protocol: A multi-modal, physician-centered intervention to improve guideline-concordant prostate cancer imaging

**DOI:** 10.1186/s13063-021-05645-3

**Published:** 2021-10-18

**Authors:** Danil V. Makarov, Shannon Ciprut, Matthew Kelly, Dawn Walter, Michele G. Shedlin, Ronald Scott Braithwaite, Craig T. Tenner, Heather T. Gold, Steven Zeliadt, Scott E. Sherman

**Affiliations:** 1 Department of Urology, New York University School of Medicine, 227 E 30th St, 617 L, New York, NY 10016 USA; 2 Department of Population Health, New York University School of Medicine, 227 E 30th St, 617 L, New York, NY 10016 USA; 3 VA New York Harbor Healthcare System, New York, USA; 4 Robert F. Wagner Graduate School of Public Service, New York University, New York, USA; 5 Perlmutter Cancer Center, New York University School of Medicine, New York, USA; 6 NYU Rory Meyers College of Nursing, New York, NY USA; 7 Department of Medicine – General Internal Medicine, New York University, New York, NY USA; 8 Health Services Research and Development, Department of Veterans Affairs Medical Center, Seattle, WA USA; 9 Fred Hutchinson Cancer Research Center, Seattle, WA USA

**Keywords:** Prostate cancer, Staging imaging, Guidelines, Implementation

## Abstract

**Background:**

Almost half of Veterans with localized prostate cancer receive inappropriate, wasteful staging imaging. Our team has explored the barriers and facilitators of guideline-concordant prostate cancer imaging and found that (1) patients with newly diagnosed prostate cancer have little concern for radiographic staging but rather focus on treatment and (2) physicians trust imaging guidelines but are apt to follow their own intuition, fear medico-legal consequences, and succumb to influence from imaging-avid colleagues. We used a theory-based approach to design a multi-level intervention strategy to promote guideline-concordant imaging to stage incident prostate cancer.

**Methods:**

We designed the Prostate Cancer Imaging Stewardship (PCIS) intervention: a multi-site, stepped wedge, cluster-randomized trial to determine the effect of a physician-focused behavioral intervention on Veterans Health Administration (VHA) prostate cancer imaging use. The multi-level intervention, developed according to the Theoretical Domains Framework (TDF) and Behavior Change Wheel, combines traditional physician behavior change methods with novel methods of communication and data collection. The intervention consists of three components: (1) a system of audit and feedback to clinicians informing individual clinicians and their sites about how their behavior compares to their peers’ and to published guidelines, (2) a program of academic detailing with the goal to educate providers about prostate cancer imaging, and (3) a CPRS Clinical Order Check for potentially guideline-discordant imaging orders. The intervention will be introduced to 10 participating geographically distributed study sites.

**Discussion:**

This study is a significant contribution to implementation science, providing VHA an opportunity to ensure delivery of high-quality care at the lowest cost using a theory-based approach. The study is ongoing. Preliminary data collection and recruitment have started; analysis has yet to be performed.

**Trial registration:**

CliniclTrials.gov NCT03445559. Prospectively registered on February 26, 2018

## Background

Prior to the widespread adoption of prostate-specific antigen (PSA) screening, most incident prostate cancer cases presented as advanced stage disease. In the PSA era, over 90% of incident prostate cancers are localized, obviating the need for routine imaging with computerized tomography (CT), magnetic resonance imaging (MRI), or radionuclide bone scan. Studies have estimated that 99% of men with low-risk incident disease do not benefit and are actually harmed by such imaging. In a VA cohort of 519 men with low-risk prostate cancer, none were found to have positive findings on bone scan [1], balanced against the relatively low sensitivity of bone scans [2]. Recognizing these trends, numerous professional societies issued prostate cancer imaging guidelines in an effort to curb overuse of imaging.

In spite of established staging guidelines, many patients undergo improper imaging. Imaging rates among men with low-risk prostate cancer have been reported to be 19–74% in a community cohort, 10–48% in a SEER-Medicare cohort, and 41% in VHA. While these rates of inappropriate use are high, there is also a significant underuse of imaging among men with high-risk disease. In a SEER-Medicare cohort of men with high-risk cancer, 70–75% underwent bone scan and 57–58% underwent CT for a total rate of 66% receiving guideline-concordant appropriate imaging; within VHA, there is still only a 70% rate appropriate imaging for men with high-risk prostate cancer.

Physicians make decisions using factors outside of those published in guidelines. Increasing Gleason score, PSA and clinical stage have all been found to be associated with greater imaging utilization even within risk groups, suggesting unnecessary over-consideration of disease severity [3, 4]. Qualitative data confirm the presence of physician-driven barriers to guideline-concordant imaging. VHA prostate cancer patients are more concerned with treatment than imaging and “trust their doctor” to make decisions [5]. All urologists believed clinical guidelines improve quality and cost of care, yet many ignored guidelines due to fear of missed pathology, trust in their intuition, or fear of litigation. These findings suggest a physician-targeted intervention would be an optimal strategy to encourage appropriate imaging.

A national level implementation program initiated in 2000 [3] by the National Prostate Cancer Register (NPCR) of Sweden [6–8] established an audit and feedback program to generate hospital-level reports of the frequency of inappropriate imaging use among patients with low-risk prostate cancer and a physician education program [6, 8, 9]. Imaging use decreased among men with low-risk prostate cancer [3]; however, imaging rates also declined among men in the high-risk category (63 to 47%). Miller et al. similarly describe a decline in imaging rates among men with low-risk prostate cancer attributed to a small-scale audit and feedback and physician education intervention undertaken within a quality-improvement consortium [10, 11]. For both interventions, inappropriate imaging of low-risk patients declined significantly but so did appropriate imaging among high-risk patients. Neither study included a control group; thus, it is impossible to determine causality. The associations described in these analyses could have been affected by unmeasured confounding or secular trends, unrelated to any intervention.

This study seeks to describe and analyze the implementation of the Prostate Cancer Imaging Stewardship (PCIS) intervention: a multi-site, stepped wedge, cluster-randomized trial to determine the effect of a physician-focused behavioral intervention on Veterans Health Administration (VHA) prostate cancer imaging use. This theory-based intervention builds on prior work identifying barriers to guideline-concordant prostate cancer imaging [4, 12] and addresses these at three levels: individual, facility, and system. The team will assess the intervention’s cost impact and providers’ experiences in preparation for a subsequent large-scale VHA implementation project optimizing the operational effectiveness of prostate cancer imaging across VHA.

## Methods/design

### Objectives

We will assess imaging rates 6 months prior (3 months prior for the first site) to the intervention and 3 months following the intervention. The study’s specific aims seek to understand the effects of the intervention on (1) facility-level prostate cancer imaging rates, (2) physician experience with and perceptions of the intervention and its implementation, and (3) the costs of both implementing the intervention and affecting change in imaging use.

- Aim 1: To determine whether a multi-modal, physician-focused behavioral intervention can improve facility-level guideline-concordant utilization of prostate cancer imaging.
- Aim 2: To use mixed methods to explore physician influence on guideline-concordant imaging.
- Aim 3: To determine the cost and cost impact of a physician-focused behavioral intervention to improve guideline-concordant prostate cancer imaging

Study setting: 10 VHA sites with high volume of prostate cancer cases and varying levels of imaging utilization.

### Study design

PCIS aims to utilize both quantitative and qualitative methods to evaluate the combination of our three evidence-based, multi-level interventions for improving the rates of guideline-concordant prostate cancer imaging at VHA. This theory-based strategy was developed based on preliminary data exploring barriers and facilitators to guideline-concordant prostate cancer imaging [5] which were subsequently mapped to effective behavior change interventions [13].

Using a stepped wedge cluster-randomized design, the first time point will be a baseline measurement, where none of the study sites have yet initiated the intervention. This study proceeds as a single direction cross-over randomized trial where every site serves, at some point, as both control and intervention [14]. This allows for a fair and accurate assessment of baseline imaging measures at each site.

**Fig. 1:**
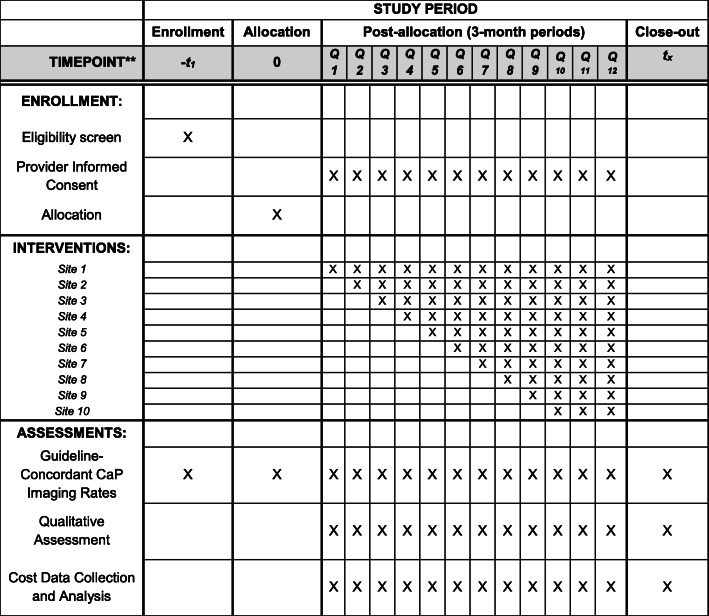
Prostate Cancer Imaging Stewardship (PCIS) intervention implementation schedule at 10 VHA study sites

As the study progresses, the time at which each site initiates implementation of the intervention is randomized via a simple random number generator and concealed in sealed envelopes until 3 months before intervention implementation date. Due to the staggered nature of the intervention, the site randomized to receive the intervention first will be ongoing for 33 months and the last site will receive a 6-month intervention (Fig. 1). The 6-month minimum duration of each intervention component is consistent with prior implementation literature [15–17]. A stepped wedge design is particularly useful for community scale interventions (e.g., a Clinical Order Check) and additionally for other financial, logistic, or ethical reasons [18]. Guaranteed access to the intervention has been a powerful recruitment tool.

### Provider eligibility

- Inclusion criteria
  - Urology chiefs and attending urologists employed through the VA (full or part time) at one of the 10 participating sites; physician assistants and nurse practitioners employed through the VA at one of the 10 participating sites that work in the respective urology clinics
  - Providers may be any gender or race/ethnicity
  - Qualitative portion only: urology chiefs and/or frontline staff physicians; participating PAs and NPs having cared for at least 5 men with incident prostate cancer within the previous 6 months
- Exclusion criteria
  - Urology residents
  To avoid potential coercion, urology residents are excluded from the study due to the hierarchical culture of surgical training programs in addition to preliminary findings that imply that residents adhere strictly to attending preference [19].

Patients are not directly recruited into the study. We have obtained a waiver of HIPAA authorization and informed consent to analyze electronic health records of patients at the 10 participating sites.

### Conceptual framework

To frame implementation and to inform the sustainability and dissemination of our findings, we used the Consolidated Framework for Implementation Research (CFIR), a compilation of existing implementation theories offering an overarching typology to understand what works in which setting and why. CFIR is composed of 5 domains: Intervention characteristics, Inner Setting, Outer setting, Individuals Involved, and Implementation Process. Each domain has within it between 4 and 12 constructs. The domains and constructs most relevant to this project include Intervention Characteristics (Evidence Strength & Quality, Trialability, Adaptability, Complexity, and Cost), Inner Setting (Networks, Culture), Outer Setting (Peer Pressure, External Policies), Individuals (Knowledge, Self-Efficacy, Individual stage of change), and Process (Planning, Engaging, Executing, and Evaluating). Qualitative analysis in aim 2 will use a CFIR-based interview guide to explore participant experience and guide subsequent dissemination.

### Intervention components

The component interventions of PCIS were developed in preliminary work based on Theoretical Domains Framework and the Behavior Change Wheel (BCW) [20].

#### Audit and feedback

Audit and feedback is an effective, individual-level intervention for changing healthcare provider behavior, resulting in small but potentially clinically important benefits [16, 21]. Audit and feedback addresses the intervention functions of education, persuasion and incentivization, all of which are important for addressing beliefs about capabilities and consequences, knowledge, and social influence determined to be significant in our preliminary work [4, 22]. We will provide quarterly feedback for both low and high-risk guideline-concordance for prostate cancer staging imaging to every participant at each study site. An attribution of guideline concordant or guideline discordant will be applied to each appointment and imaging test ordered by enrolled providers. Guideline concordance is determined according to version 2018.2 of the National Comprehensive Cancer Network’s (NCCN) imaging guidelines for staging prostate cancer [23]. Feedback will be given to each provider individually and will include his or her individual-level data as well as aggregated data for the local institution (including both participants and non-participants) and VHA as a whole as collected from local clinics and CDW data and validated centrally by study staff via manual chart review. The data will be reviewed during a brief group meeting between the Site PI and participants at the clinic, arranged at their discretion, and will include specific recommendations for overall performance improvement from the Site PI for the site as a whole. Site PIs will receive de-identified individual provider imaging rates, so long as there are more than three participants enrolled at that site. Individual participants will only see their own individualized reports. Participants who are not serving as Site PI will not see their colleagues’ individual-level data and will not be aware of any other individual enrollment status. There is no anticipated harm and compensation for trial participation.

#### Academic detailing

Academic detailing (also known as educational outreach) is an individual and facility-level intervention consistently shown to affect provider behavior [15, 21]. This strategy addresses the intervention functions of persuasion, modeling, and education which are effective methods for affecting behaviors driven by beliefs about capabilities, knowledge, social influences, beliefs about consequences, and environmental context and resources [4, 22, 24]. The academic detailing sessions will take place at the initiation of the intervention and then regularly thereafter as determined by the local investigator team throughout the intervention period. The initial session will be performed by the study PI and project manager along with the local site PI; subsequent sessions, to encourage sustainability, will be performed by the local site PI. During the group meeting, the representatives from the investigator team will follow a script explaining that the visit is part of an experimental program to provide physicians and providers with up-to-date, unbiased information about imaging to stage prostate cancer [15, 25]. The representatives will review summary information from the NCCN and AUA prostate cancer imaging guidelines and encourage the provider participants to modify their ordering behavior to comply with those guidelines or reinforce the behavior of those already in compliance [26]. Appeals based on fear or coercion will be avoided. Improvement of clinical care will be emphasized above cost considerations. Providers will be encouraged to participate in the educational exchange and to discuss specific problem cases. Summaries of the guidelines and their URLs will be left with providers. The agenda for the visit will include:

1. Review prostate cancer imaging guidelines
2. Demonstrate the clinical reminder
3. Describe the audit and feedback program
4. Present local and national imaging rates
5. Answer any questions

Subsequent academic detailing sessions may occur in-person, by phone, or over e-mail, at the discretion of the site-PI. Sessions may take place during regularly scheduled urology section meetings.

#### Clinical Order Check

**Fig. 2:**
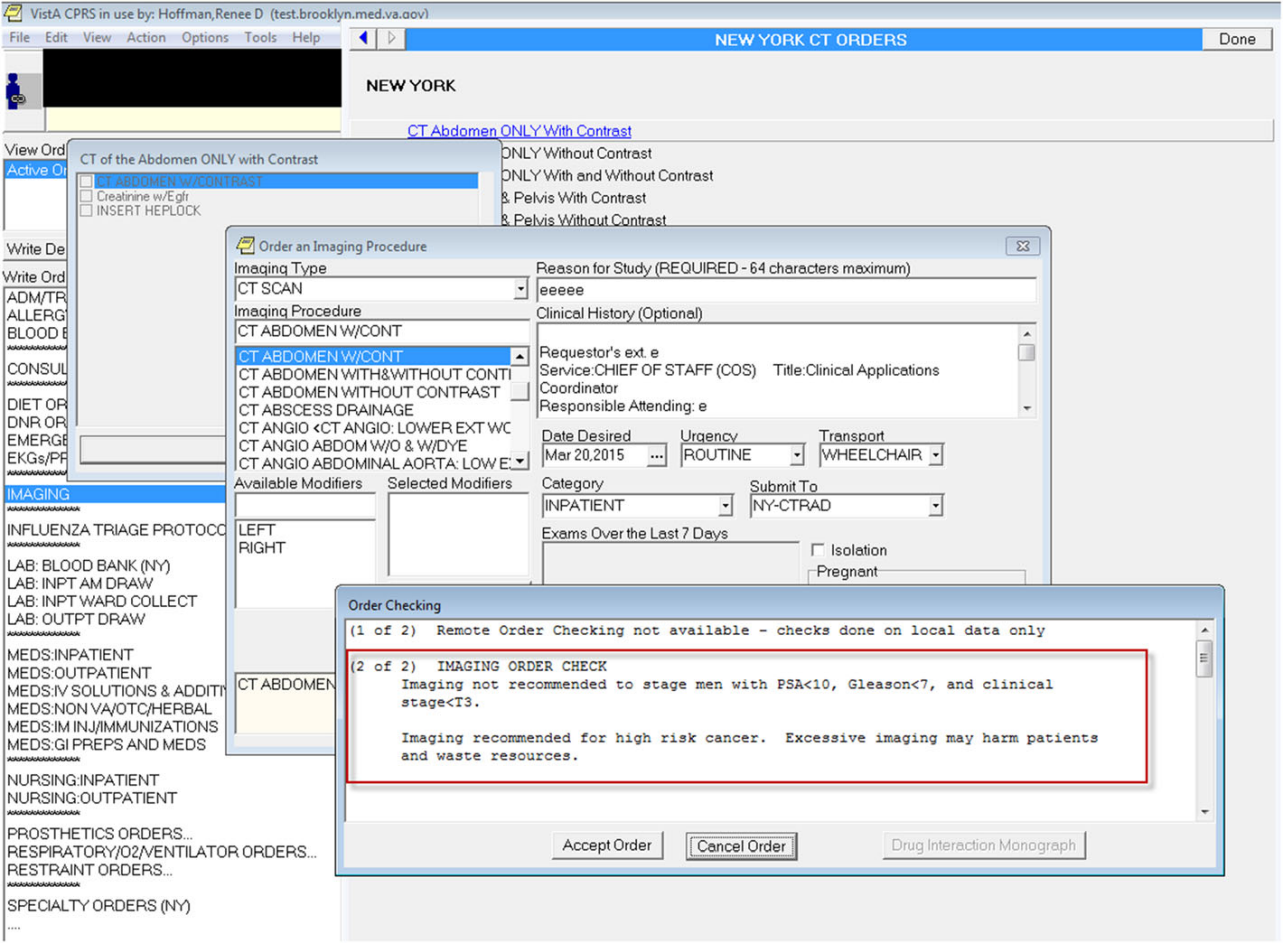
CPRS screen capture of Clinical Reminder Order Check (CROC)

A Clinical Order Check is an evidence-based, systems-level method to affect behavior change [17, 27–29]. It addresses the intervention functions of education, enablement, and incentivization which are effective methods to change behaviors driven by beliefs about capabilities, knowledge, social influences, beliefs about consequences, and environmental context and resources; all domains previously established to be associated with prostate cancer imaging [5, 30, 31]. All VA facilities currently use locally adapted clinical reminders. We will adapt the Order Check currently in use at VA New York Harbor Healthcare System (VANYHHS – implemented by Drs. Makarov, Sherman and Tenner) for implementation at other study sites with guidance from their local Clinical Advisory Committees. This strategy is technologically simple, straightforward, and considered to be a best practice within the VA IT community [32]. As at VANYHHS, the reminder will be self-explanatory and non-intrusive to workflow. Reminder specifics include:

1. Selection criteria: The Clinical Order Check will appear when a patient has the following characteristics:
  - Male sex
  - New diagnosis of prostate cancer within 6 months of the current date
  - Serum PSA < 20 ng/mL. Those with higher PSAs all require imaging.
  - Imaging modality: Provider selects: bone scan or axial imaging of abdomen or pelvis
2. Content: Based on consultations with local physician leaders and administrators, we agreed on the following text for the Order Check: “Imaging not recommended to stage men with PSA < 10, Gleason< 7, and clinical stage < T3. Imaging recommended for high-risk cancer. Excessive imaging may harm patients and waste resources” (Fig. 2). Local site advisory committees may modify this text according to their practice needs and culture.
3. Opt out: Providers may override the recommendation against ordering and will be asked to explain their reasons for doing so. The local IT representative, as part of the Local Clinical Advisory Committee, will be able to pull these responses from CPRS, in addition to the number of times the Opt Out option was utilized during the intervention period.

This feature will be implemented at the institution-level discrimination among providers by enrollment status. Providers who are NOT participating in this study need simply to click (“x”) out of the pop-up notification to proceed with their original order. There will be no special criteria for discontinuing or modifying allocated interventions. Academic detailing sessions and subsequent quarterly meetings will review the intervention components and assure provider engagement and adherence to protocols. Implementing PCIS will not require alteration to usual care pathways and these will continue for in the included trial clusters.

### Evaluation

#### Quantitative measures

Three validated instruments will be administered prior to the initiation of the intervention to all subjects at participating sites as one collective pre-intervention survey: The Organizational Readiness to Change Assessment (ORCA scale) [33], Evidence Based Practice Attitudes Scale (EBPAS) [34], and a Self-Assessment of Contextual Fit [35], along with a demographic questionnaire. The first set of instruments assesses organization strengths and weaknesses to support implementation of evidence based practices as well as a provider’s feelings towards adopting new practices. The CFIR constructs captured through this measurement are as follows: evidence strength and quality, structural characteristics, characteristics of the individual, networks and communication, culture, compatibility, incentives and rewards, goals and feedback, leadership engagement, planning, and reflecting. After the intervention, another survey consisting of two additional validated instruments will be distributed to all participants: the Adoption of Information Technology Innovation-Compatibility Subscale [36], Level of Success Instrument [37], and a modified Self-Assessment of Contextual Fit [35]. These will measure the degree to which providers feel new technologies are compatible with their work, the degree to which the adoption the innovation was successful, and the contextual fit of the intervention within the clinical environment. ORCA will also be administered again within the post-intervention survey. The CFIR constructs of compatibility and penetration will be measured through these tools.

#### Qualitative component

We will be conducting a qualitative analysis to assess participant opinions of the intervention and how those perceptions relate to prostate cancer imaging use, among a subsample of the enrolled providers (*N* = 25–40). At the end of the intervention we will administer one-on-one, in-depth semi-structured interviews between a participant and a member of the research team in person or by phone, using a CFIR-based interview guide. We will also interview participating site-PIs and urology chiefs.

### Analysis plan

Statistical analyses will be performed using the SAS 9.1.3 Service Pack 4 statistical package (SAS Institute, Cary, NC) and Excel 2010 (Microsoft Corporation, Seattle, WA). Qualitative data will be analyzed using NVivo 10 (QSR International). A private, password protected REDCap project will be created for this study. Study team members will ascertain the input data from the questionnaire as well administered to participants. Site-specific results will be de-identified and will not be shared with anyone beyond the central research team. Specifically, only aggregated data will be shared with Chief or Site PI. Site-specific information will be collected for analysis purposes only; results will be reported as aggregate. No interim analyses will be performed, as there are no anticipated problems that are detrimental to the participants.

### Quantitative analysis—aim 1

Quantitative data for this study is from VHA’s Corporate Data Warehouse (CDW) and local clinic staff. VINCI, VHA’s secure data environment, will be used to identify prostate cancer patients in CDW as well as their imaging tests, demographic information, and clinical history. The VINCI team will extract required data from CDW tables and create a work environment for our team on secure VINCI servers in Salt Lake City. Data from the local clinic record will be accessed using CAPRI to generate timely data for Audit and Feedback. In further efforts to avoid delays due to any lags in CDW data availability, the local IT specialist will run a reoccurring monthly report in CPRS in an attempt to identify patients diagnosed with prostate cancer in real-time. The results will yield patient name, SSN, and presumed date of prostate cancer diagnosis. The IT specialist will send the results of these queries to the central research team via PKI-encrypted email on VA outlook when requested. The central research team will verify and validate this data using concentrated chart review within CAPRI. Patients with missing data that prevents risk-categorization via NCCN guidelines [23] will be excluded from the study.

The sample size of 10 study sites was determined to ensure appropriate power for our primary outcome: differences in the rate of inappropriate prostate cancer imaging. For the sample size calculation of a stepped wedge trial, the key variables are the number of clusters (i.e., sites), *I*; the number of distinct time points or intervals being compared, *T*; and the number of outcome observations per time point, *N* (i.e., the number of individual patients with the outcome per cluster, per time interval) [14]. We assume the model, *Y*_*ij*_ = *μ* + *α*_*i*_ + *β*_*j*_ + *X*_*ij*_*θ* + *e*_*ij*_, where *α*_*i*_ is a random effect for cluster *i* such that *α*_*i*_~*N*(0, *τ*^2^), *β*_*j*_ is a fixed effect corresponding to time interval *j*, *X*_*ij*_ is an indicator of whether the intervention has been implemented in cluster *i* at time *j* (1 = intervention; 0 = control), *θ* is treatment effect and *e*_*ij*_ = ∑_*k*_*e*_*ijk*_/*N* are independent and identically distributed *N*(0, *σ*^2^) and *σ*^2^ = *σ*^2^_*e*_/*N*. Let*Y*_*ij*_ be the mean for cluster *i* at time *j*. Assume testing the hypothesis *H*_0_ : *θ* = 0 versus *H*_*A*_ : *θ* = *θ*_*A*_, where *θ*_*A*_ is the treatment effect size. The approximate power for conducting a 2-tailed test of size alpha is $power=\Phi \left(\left({\theta}_A/\sqrt{Var\left(\tilde{\theta}\right)}\right)-{Z}_{1-a/2}\right)$ where Φ is the cumulative standard normal distribution function, *Z*_1 − *a*/2_ is the (1 − *a*/2)*th* quantile of the standard normal distribution function and $\hat{\theta}$ is the estimated effect size. The estimated number of patients exposed to the intervention will be 750, compared to 750 control patients, which will provide sufficient power for even modest improvements in imaging rates among low-risk men. Assuming 10 time periods (Q2-Q11), an estimate for baseline imaging among men with low-risk prostate cancer of 40% in the usual care group, and a decrease to 20% guideline-discordant imaging (absolute difference of − 0.20), 10 clusters (sites), 15 patients with low-risk prostate cancer per quarter × 10 study sites × 10 quarters is estimated to impact 1500 total patients (a conservative estimate based on analysis of VINCI data), an alpha of 0.05, and a coefficient of variation of 0.40, accounting for clustering, we would estimate having a power of > 0.999. This is a conservative estimate in terms of the expected effect of the intervention on prostate cancer imaging rates and assumes a high coefficient of variance with outcomes highly correlated with site. A more conservative post-intervention rate of imaging of 28.7% would reduce power to 0.80.

Similarly, we estimate that we will have sufficient power to detect increases in appropriate imaging among men with high-risk prostate cancer. Assuming 10 time periods (Q2-Q11), an estimate for baseline appropriate imaging of 66% in the usual care group, and an increase to 86% (for an absolute difference of + 0.20), 10 clusters (sites), 5 at patients with high-risk prostate cancer per quarter (a conservative estimate based on analysis of VINCI data), alpha of 0.05, a coefficient of variation of 0.40, we estimate power of 0.89.

We will also perform exploratory, individual-level analyses. If providers consent to participate in the study, then these imaging outcomes will be linked to their survey and qualitative data. For non-participants, imaging patterns alone (with no additional data) will be analyzed in a de-identified manner.

### Qualitative analysis—aim 2

Qualitative data will add depth and detail to our analysis, complementing the other findings to explain and illustrate quantitative results. In-depth interviews at the conclusion of the study will explore providers’ experiences with the intervention and explain the important implementation-related domains from CFIR [30]. The exploratory nature of this component will permit the identification of new ideas and inform the generation of inductive hypotheses regarding factors motivating guideline-concordant imaging. We will also ask a short series of questions exclusively to all Urology Chiefs and Site PIs (*N* = 20) to explore institutional and managerial perspectives of implementation and attitudes towards intervention sustainability [38]. Data gathered will be critical to the VAMC nationwide dissemination plan. We anticipate recruiting a subset of approximately 20–30 frontline providers across the 10 participating study sites in order to reach theoretical saturation. There are 79 practicing urologists at all 10 sites (10 chiefs and 67 frontline providers) so we anticipate no difficulties in reaching our recruitment goal.

### Cost analysis—aim 3

Clinical care cost data will be accessed primarily through the Health Economics Resource Center (HERC) Average Cost File. HERC has created estimates of the cost of all VA health care encounters that have taken place since October 1, 1998. These data are accessible approximately 6–8 months following end of the fiscal year. These data will allow for comparable standardized prices to be applied across all VHA facilities for all follow-up care activities. Cost data will also be obtained from billable private insurance claims. VHA’s Medical Care Recovery Program attempts to collect for care performed at VHA when a VA user has private insurance. We will identify subjects in our cohort who have billable private insurance and flag these subjects for exclusion in sensitivity analyses as they may be likely to be more reliant on community care than VA-users without private billable insurance. Preliminary analysis in VISN20 identified that out of a cohort of 260,743 subjects, 17,141 (6.6%) had billable insurance. This variable is available in the CDW.

We will also estimate the cost of implementing the intervention using a societal approach. Costs for central research staff will be estimated using weekly time audit logs to attribute time devoted towards implementation of the intervention, excluding other research and administrative tasks from the calculation. For local site staff and practitioners’ costs will be estimated using the approved project budget. We will categorize study tasks into costs associated with implementation and with research. These methods allow for the documentation of organizational costs associated with the implementation effort itself, not just the costs associated with changes in patient care or research tasks that would not need to be repeated if the intervention were to be implemented in a new clinical setting. Data sources used for estimating costs will include interviews, surveys, project schedules, project budgets and cost records, and government salary information.

### Outcomes

The study’s primary outcomes determined from the above data sources are as follows:

Specific aim 1

1. Facility-level utilization of bone scan or abdominal/pelvic CT or abdominal/pelvic MRI among men with newly diagnosed, low-risk prostate cancer. (“Inappropriate Imaging”)
2. Facility-level utilization of bone scan or abdominal/pelvic CT or abdominal/pelvic MRI among men with newly diagnosed, high-risk prostate cancer. (“Appropriate Imaging”)
  Specific aim 2
3. Provider-level utilization of bone scan or abdominal/pelvic CT or abdominal/pelvic MRI among men with newly diagnosed, low-risk prostate cancer. (Inappropriate Imaging)
4. Provider-level utilization of bone scan or abdominal/pelvic CT or abdominal/pelvic MRI among men with newly diagnosed, high-risk prostate cancer. (Appropriate Imaging)
5. Provider attitudes regarding prostate cancer imaging guidelines and the behavioral intervention

Specific aim 3

1. Net cost, including costs of facility-level workforce and imaging technology, of implementation of physician behavioral intervention in VHA.

Facility-level descriptors will be obtained from the 2009 VHA Oncology Services Survey. As part of the Office of Patient Care Services initiative to conduct systematic program reviews, Oncology Services conducted a survey of cancer care services in VHA. This study is exempt from Data Safety and Monitoring Board review per the study sponsor VA HSR&D. The sponsor played no part in study design; collection, management, analysis, and interpretation of data; writing of the report; and the decision to submit the report for publication.

## Discussion

### Innovation and potential impact

PCIS is an opportunity to leverage VHA’s state-of-the-art, integrated healthcare delivery system to implement a carefully designed, theory-based behavioral intervention to reduce harmful, inappropriate care, increase appropriate care to those who truly need it, and simultaneously save money for the healthcare system. This study is designed to make a significant contribution to implementation science, providing VHA an opportunity to ensure delivery of high quality care at the lowest cost using a theory-based approach.

### Harms

Adverse events and serious adverse events are not likely to occur due to the nature of this intervention. The intervention does not entail greater than minimal risk to either provider participants or their patients. If a reportable event such as an unanticipated problem or protocol deviation should occur among the research team, the PI will be notified immediately.

## Data Availability

The datasets generated and/or analysis during the current study are not publicly available due to Veteran PHI involved and VA data restrictions.

## Abbreviations

CDW: Corporate Data Warehouse
CFIR: Consolidated Framework for Implementation Research
CPRS: Computerized Patient Record System
ORCA: Organizational Readiness for Change Assessment
LSI: Local Site Investigator
VHA: Veteran’s Health Administration
VAMC: Veterans Affairs Medical Centers
